# The impact of environmental literacy on the health level of rural residents: evidence from the mountainous areas of Sichuan, China

**DOI:** 10.3389/fpubh.2024.1465483

**Published:** 2024-12-30

**Authors:** Yue Shui, Yingjie Yang, Shaoquan Liu

**Affiliations:** ^1^College of Geography and Planning, Chengdu University of Technology, Chengdu, China; ^2^Institute of Mountain Hazards and Environment, Chinese Academy of Sciences, Chengdu, China

**Keywords:** environmental literacy, rural residents, health level, mountainous areas, China

## Abstract

**Introduction:**

Environmental pollution and health issues are hot topics of discussion in modern society. However, there is a lack of research from the perspective of subjective factors such as environmental protection to study the impact of environmental literacy on health, especially in rural areas.

**Methods:**

First, through field research in the mountainous rural areas of Sichuan Province, 396 data points were collected. Based on the KAP model, we constructed an interactive impact mechanism model for the health of rural residents. We used chi-square tests and t-tests to examine the relationship between the background characteristics of rural residents and environmental protection. All environmental literacy variables were classified into strong and weak observational variables, and a multiple linear regression model was employed to explore the impact mechanism of environmental literacy (divided into environmental protection awareness, attitudes, behaviors, and perceptions of environmental pollution) on the health of rural residents.

**Results and discussion:**

(1) Young village officials with higher education levels are more likely to have stronger environmental literacy. (2) The health status of rural residents is positively affected by their positive attitude towards environmental protection and negatively affected by their environmental protection behaviors. (3) Age, gender, marital status, and party membership have a significant impact on the health of rural residents. The results of this study can enhance the attention to the living environment and health in rural areas, and provide a scientific basis for improving the environmental literacy and health level of rural residents.

## 1 Introduction

Under the pressing concerns of global environmental degradation, governmental bodies and associated institutions are progressively intensifying environmental propaganda, thereby further advocating for the adoption of energy-saving and emission-reduction concepts by the general public. In 2021, China formally proposed the strategic goals of “carbon peak and carbon neutrality.” The “dual carbon” strategy requires the participation of all people, which naturally imposes higher demands on the environmental literacy of the public (1). Therefore, against the backdrop of the “dual carbon” strategy, the status of environmental literacy among Chinese residents becomes particularly noteworthy. Because humans are inextricably linked to the environment, social knowledge, as well as that of environmentally significant behavior (53, 54) and avenues for addressing environmental issues, is necessary for environmental programming to achieve its goals. Given this general situation, most residents have developed a considerable degree of environmental literacy. An increasing number of rural residents recognize the importance of the ecological environment and choose to recycle waste as fertilizer, adopting green and sustainable methods for agricultural planting (2, 3). Under the relatively isolated conditions of mountainous areas, the level of environmental awareness among rural residents varies, as does the amount and choice of chemical fertilizers and pesticides, resulting in different levels of pollution (4). If this continues long-term, it will further exacerbate pollution levels and produce harmful consequences. Therefore, it is of practical significance to place emphasis on the awareness of environmental protection, lifestyle, and health status of rural residents.

In 1968, the American scholar Roszak first proposed the concept of “environmental literacy (EL).” As research has deepened, the scope of the field has continuously expanded. In addition to measuring environmental literacy, the complex relationships between the components of environmental literacy have increasingly become the focus of research (55, 56). We define EL as knowledge of and dispositions (e.g., environmental identity and self-efficacy, connection to nature) towards environmental (social-ecological) systems, practices (e.g., identifying issues, creating possible solutions) one uses while engaging with those systems, and the behavior that results (5). Environmental literacy includes multiple elements such as environmental knowledge, environmental awareness, environmental attitudes, environmental behaviors, and so on (6–10). Recent research has indicated that environmental literacy has the potential to influence various aspects of rural residents’ livelihoods and productivity (11). For instance, a heightened awareness of environmental protection positively impacts rural residents’ responses to policies, while also influencing their environmental behaviors in a positive manner. Scholars have presented three dimensions of rural residents’ environmental literacy, namely: awareness of environmental issues, attitudes towards environmental protection, and tolerance for environmental pollution. Through studying the relationship between rural residents’ environmental literacy and the enhancement of their living environment, researchers have discovered a significant influence of environmental literacy on their improvement behaviors (12). Teng et al. (13) found that farmers’ energy saving emotion and ecological values were the main factors affecting energy saving behavior. However, it should be noted that certain studies have identified a discrepancy between environmental literacy and actual pro-environmental behaviors among rural residents (14, 15). Therefore, further in-depth studies are required to fully comprehend the tangible impact of environmental literacy on the production-related activities of rural residents.

Currently, the literature pertaining to the health of rural residents predominantly adopts a medical standpoint, focusing primarily on the determinants influencing their health status. Within this domain, subjective psychological factors have gradually garnered attention. Broadly speaking, health concerns are increasingly situated within the purview of natural, economic, and social systems, and analytical approaches are characterized by a more holistic and systemic utilization of concepts and methodologies (16). Both domestic and international scholars dedicate their attention to the determinants of health, whereby foreign scholars generally regard that income and wealth bear no direct association with health. Instead, marital status, employment, proficiency in health-related knowledge, environmental conditions, social relations, educational quality, community standing, and self-awareness exhibit stronger correlations with health status (17, 18). The study into factors impacting the health degree of rural residents has been explored from diverse angles, chiefly encompassing regional economic and social development, the economic and cultural circumstances of rural residents, and the capacity of regional health services (19, 20). Notably, certain scholars have delved into the factors influencing self-reported ailments among residents residing in impoverished rural areas, deducing that health consciousness, economic circumstances, and psychological stress serve as predominant determinants (21, 22). Existing research on the health of rural residents primarily emanates from a medical perspective, with a conspicuous dearth of an encompassing empirical analytical framework (23).

In addition, the measurement of the health degree in the research has also changed from a single physiological or psychological indicator to comprehensive physiological, social and psychological indicators, and it will be more scientific to transform these indicators into a relatively objective health index for health research. The prevailing quantitative research on the health status of rural households primarily employs health degree as independent variables rather than dependent variables. In addition, the utilization of distinct models and indicators has led to substantial variations in the derived conclusions. Moreover, the factors under consideration have predominantly encompassed objective variables, such as age, gender, and the natural and economic circumstances of the rural community. Conversely, scant attention has been given to investigating the influence of environmental literacy factors, including ecological awareness and environmental protection, on the health of rural residents. Furthermore, a theoretical framework for such analysis is notably lacking. Consequently, this study has the potential to address gaps in the existing literature and advance the research framework pertaining to the health degree and lifestyles of rural residents.

The Knowledge-Attitude-Practice (KAP) model, which originated from learning theory and diffusion of innovation theory, provides a theoretical framework for this study (24). In this study, the KAP model was used to examine the environmental literacy of rural residents. The KAP model suggests that knowledge is an antecedent to attitude formation and drives practical behaviors through attitudes. In this context, knowledge is the awareness or understanding of information, attitude indicates a positive or negative evaluation of a goal, and behavior refers to regular activities carried out in the face of different problems. The model has been widely used in social research fields such as family planning, public health, education, sports, etc., and its applicability in identifying cognitive differences, attitudinal barriers and individual behaviors has been well tested. As the model continues to mature, the KAP model has also been expanded to green sustainable development fields (such as sustainable community development), land use change and rural land policy practice fields (such as rural industrial land), and other research fields (24, 25).

**Figure 1:**
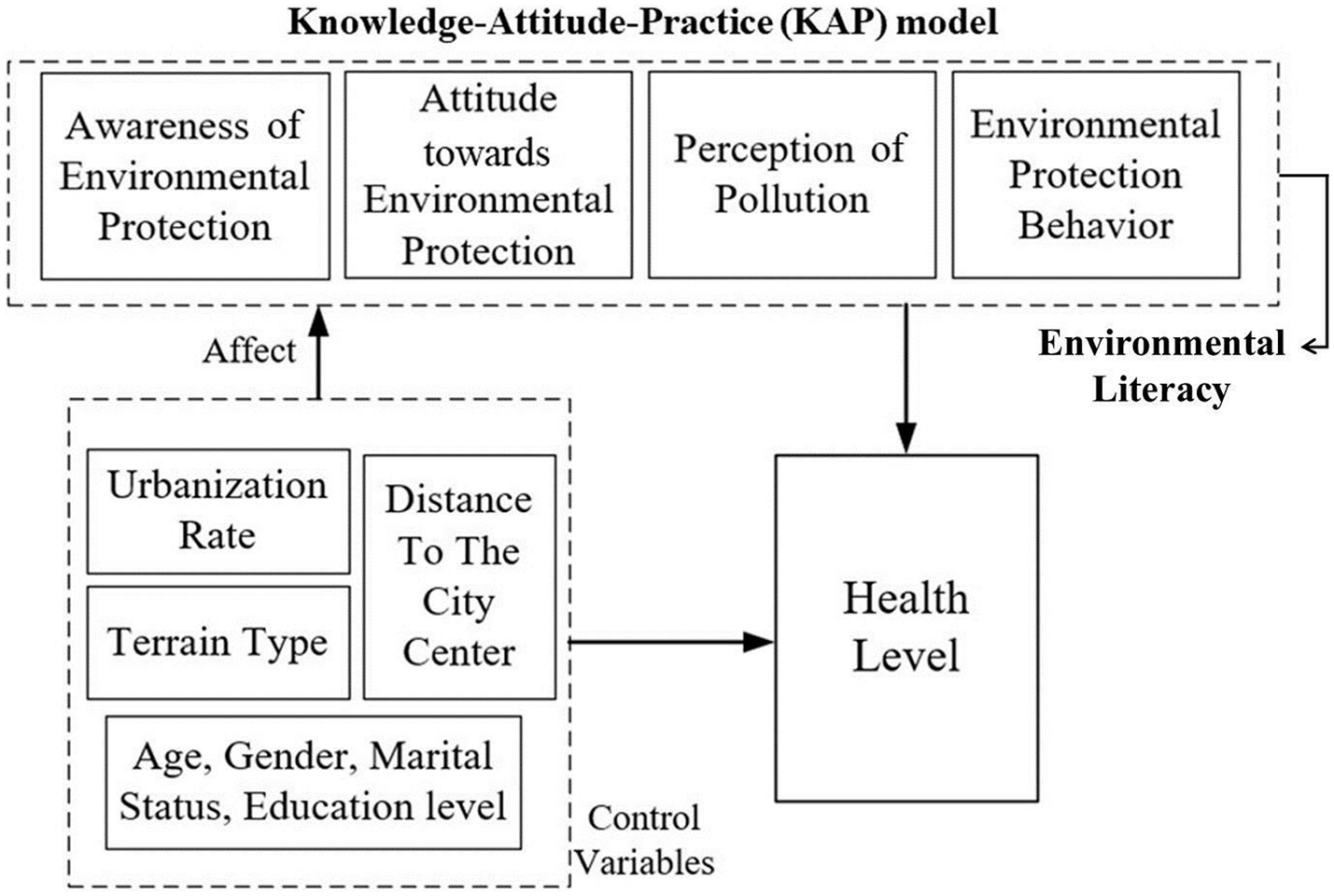
Theoretical analysis framework of the influence mechanism of farmers’ health degree.

This paper combines the KAP model and existing domestic and international studies to improve the theoretical analytical framework from the specific situation of rural residents in mountainous areas of China in order to elucidate the interactive influence mechanism of their healthiness (Figure 1). Subsequently, this study conducted research and analyses based on this framework, dividing the investigated influencing factors into environmental literacy and control variables. The environmental literacy variable adds “pollution perception” to the KAP model, which consists of four main aspects: rural residents’ knowledge of environmental protection (In this study, we use environmental protection awareness to summarize this content), attitudes towards environmental protection, perception of pollution, and participation in environmental protection behaviors. On the other hand, the control variables predominantly encompass individual factors of rural residents (e.g., age, gender, marital status, and educational level) as well as aspects related to the villages they inhabit, such as terrain type, urbanization rate, and distance to the city center. The control variables exhibit interplay and mutual reinforcement, collectively influencing both the environmental literacy and the health degree of rural residents. Simultaneously, the environmental literacy also exert a certain impact on health degree. This paper seeks to appraise the correlation between the environmental literacy of rural residents in mountainous areas with their health degree. Therefore, it establishes a theoretical analysis framework for exploring the research on health degree in relation to these factors.

## 2 Data and methods

### 2.1 Research area and data source

The data used by the paper institute comes from the questionnaire survey conducted by the research group in Sichuan Province in 2019, which covers the basic situation of farmers’ families, family production, family income and expenditure, etc. The reasons for using this data are as follows: Sichuan Province, situated in southwestern China, encompasses a total area measuring 485,000 square kilometers. Representing a prototypical mountainous province in the western part of the country, approximately 76.83% of its overall territory comprises mountainous counties (57). Simultaneously, there exists a rural population of 36.209 million individuals within Sichuan Province, accounting for 43.27% of the total populace, thus portraying it as a paradigmatic agricultural province. For the purpose of this study, the focus area encompasses the rural regions within the mountainous locales of Sichuan Province. These rural areas possess distinctive spatial attributes, including small size, elevated terrain, dispersed settlements, remote locations, seclusion, and bordering proximity. They feature a substantial proportion of illiterate and semi-literate individuals, a considerable prevalence of rural settlements lacking road connectivity, as well as a notable scarcity of medical resources available to the population (26). Due to geographical constraints, these rural areas experience relative isolation, potentially resulting in significant variations in the residents’ environmental awareness compared to other regions.

**Figure 2:**
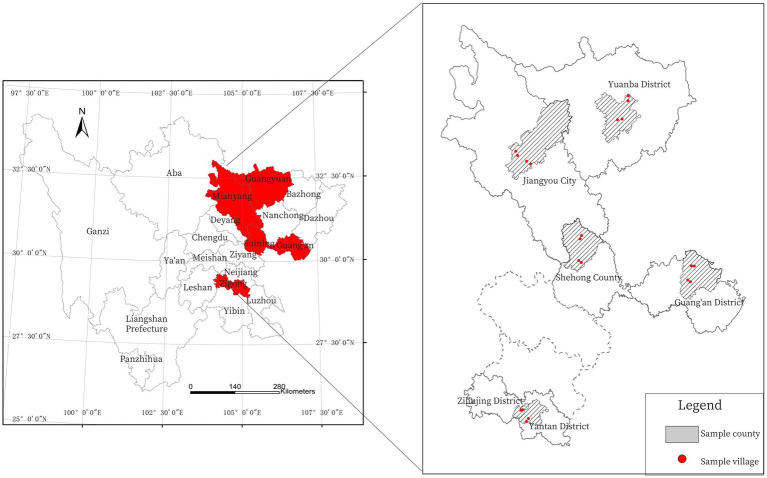
Study area (52).

Given the considerable variations in economic and social development both across different counties in Sichuan Province and within each individual county, a rigorous and systematic approach was employed to ensure the representativeness and diversity of regional characteristics (27). The survey adopts stratified sampling and equal probability random sampling to select respondents and the data is detailed and reliable (28). First, the 183 counties within Sichuan Province were classified into five groups based on per capita industrial output value. A portion of counties was randomly selected from each group, resulting in the identification of five sample areas representing varying degrees of economic development. Subsequently, employing the same methodology, each sample area was further divided into two groups. From each group, one township was randomly selected, yielding a total of two townships and ten sample townships. To ensure a balance between socioeconomic development levels and geographical distribution, two administrative villages were randomly chosen from each sample township, resulting in a total of 20 sample villages. Finally, employing the village roster and a random number table, 20 households were randomly selected from each village, resulting in a total of 400 household samples, and the corresponding questionnaire was administered. The geographical distribution of the sample villages is presented in Figure 2. Following thorough data screening and cleaning procedures, a final dataset of 396 samples was obtained.

In the sampled area, the mountainous counties were classified into two categories based on the proportion of their mountainous and hilly areas (mountain counties: mountain area ≥ 40% and hill area < 50%; hill counties: mountain area ≥ 40% and hill area ≥ 50%, or mountain area < 40%), and each sampled village was assigned a terrain type based on the classification of its respective county (29).

### 2.2 Research methods

#### 2.2.1 Measure of rural residents’ environmental literacy

With reference to the study by various researchers (12, 30) a questionnaire was meticulously devised, encompassing a comprehensive set of 15 inquiries. These questions were intended to understanding the cognizance of rural inhabitants with respect to environmental preservation, their disposition towards environmental conservation, their understanding of pollution, and their corresponding environmental protection practices (Table 1).

**Table 1:** Scale of rural residents’ environmental literacy.

	No.	Questions	Choices
Awareness of environmental protection	1.	Do you think pesticides pollute farmland?	1 = Yes; 2 = No; 3 = No idea
	2.	Do you believe that the disposal of pesticide bottles pollutes farmland?	1 = Yes; 2 = No; 3 = No idea
	3.	Do you believe that chemical fertilizers cause pollution to farmland?	1 = Yes; 2 = No; 3 = No idea
	4.	Do you believe that organic fertilizers pollute farmland?	1 = Yes; 2 = No; 3 = No idea
	5.	Do you believe that farm manure pollutes farmland?	1 = Yes; 2 = No; 3 = No idea
	6.	Do you believe that plastic mulch pollutes farmland?	1 = Yes; 2 = No; 3 = No idea
	7.	Do you believe that returning straw to the field pollutes farmland?	1 = Yes; 2 = No; 3 = No idea
	8.	Do you believe that irrigation pollutes farmland?	1 = Yes; 2 = No; 3 = No idea
Attitude towards environmental protection	1.	Are you willing to spend time to protect the environment from pollution?	1 = Yes; 2 = No
	2.	Are you willing to spend money to protect the environment?	1 = Yes; 2 = No
	3.	Are you willing to take actions to reduce environmental pollution?	1 = Yes; 2 = No
Perception on pollution	1.	Has there been an increasing trend of man-made environmental pollution in your area in recent years?	1 = No; 2 = No idea; 3 = Yes
	2.	What are the trends in the pollution of rivers, streams, and other water bodies in your area?	1 = Mitigation; 2 = Unchanged; 3 = More serious
Environmental protection behavior	1.	Have you watched or read any TV programs or books about environmental protection?	1 = Yes; 2 = No
	2.	Whether to recycle rigid plastics/plastic bags/paper waste paper?	1 = Yes; 2 = No
	3.	The frequency of Garbage classification and processing.	1 = high; 2 = low

We take environmental protection awareness as an example to elaborate on how we reduce the dimensionality of multiple items from the original scale. The definition of awareness of environmental protection is subject to varying interpretations among scholars. According to some, it encompasses a comprehensive range of social thoughts, theories, emotions, willpower, and perceptions. This multifaceted construct reflects the intricate connection between humanity and nature, embodying a novel set of values that promote the harmonious development of human and natural environment interactions (30). In contrast, alternative perspectives suggest that environmental awareness involves the acquisition and mastery of knowledge pertaining to environmental protection, as well as the cultivation of conscientiousness in guiding environmentally friendly behaviors (31). Additionally, it has been argued that environmental awareness is the subjective perception and behavioral tendency of individual social members to the relationship between human and environment, and is the result of a series of complex psychological processes (32). In accordance with related research, this paper defines that the rural residents’ awareness of environmental protection as the extent to which individuals understand the environment and the principles of safeguarding it within the context of their livelihood and production activities. It encapsulates residents’ cognizance and ongoing commitment to adjusting their production practices and social conduct in order to preserve the environment, while simultaneously fostering a harmonious equilibrium between humans and their surroundings, as well as within themselves. Environmental attitude pertains to the willingness of rural residents to actively engage in environmentally friendly practices in their daily lives, serving as an indicator of their emphasis on environmental protection. Perception of pollution corresponds to the degree of perceptiveness exhibited by rural residents towards environmental issues that result in pollution, thereby elucidating the extent of their concern regarding such matters. Lastly, environmental behavior encompasses the proactive involvement of rural residents in past environmental conservation endeavors, as well as their willingness to undertake precautionary measures to address future environmental challenges.

For the analysis of the data pertaining to the awareness of environmental protection, a factor analysis approach was employed based on the principal component analysis model. In accordance with the criterion stipulating an eigenvalue exceeding unity, two distinct common factors were identified and subsequently extracted. Subsequently, to effectuate factor rotation, the Varimax method, complemented by Kaiser Normalization, was implemented.

This study employs factor analysis to derive two factors associated with the awareness of environmental protection, as illustrated in the remaining variables presented in Table 1. To ensure accurate results, the K-means clustering technique is utilized. It should be noted that different approaches for determining the initial values of the centroids may yield dissimilar results. To minimize random classification, the class average method is first employed within the systematic clustering approach, employing Euclidean distance for clustering. Subsequently, the sample numbers represented by the leaves in the dendrogram are arranged in ascending order (from left to right). By utilizing the K-means clustering method, all variables in the questionnaire are classified into two categories pertaining to rural residents. Importantly, the mean differences between the two identified rural resident types, based on each factor and variable, have achieved statistical significance at the 99% confidence level.

The average factor scores reveal that the first type of awareness of environmental protection is predominantly characterized by negative scores (indicating a greater inclination to choose “yes” to a larger number of questions in the awareness of environmental protection scale). Consequently, this type is classified as rural residents with a “strong awareness of environmental protection.” Conversely, the factors associated with the second type predominantly exhibit positive scores, leading to their classification as rural residents with a “weak awareness of environmental protection.” By the same token, rural residents are further categorized into groups based on strong and weak environmental attitudes, strong and weak perceptions of environmental pollution, as well as strong and weak environmental behaviors.

#### 2.2.2 Measure of rural residents’ health level

The research used three indicators of self-rated health, whether you have chronic diseases diagnosed by doctors, and whether you were sick and hospitalized in the past year to measure health degree. Use factor analysis to check whether the three indicator variables are measuring a unique factor variable (health degree). The method of extracting factors is principal component factor method.

To be specific, only a strong correlation can be used for principal component analysis, and the correlation test of various indicators can be carried out. The results are shown that the value of K in the table is 0.602 > 0.5, *p* value is 0. Through the significance test, it can be the principal component analysis.

Table 2 shows the results obtained by the principal component factor method. It shows that a common factor with an eigenvalue greater than 1 is extracted, and its cumulative variance contribution rate is 55.297%, indicating that the three indicator variables are indeed measuring the only factor variable (health degree). The result of factor analysis shows that the selected three index variables can measure the “health degree” well.

**Table 2:** Principal component analysis of health level.

	Eigenvalue	Factor loading	Variance (%)	Cumulative variance (%)
X_1_ (Self-rated health)	1.659	0.472	55.297	55.297
X_2_ (Whether you were sick and hospitalized)	0.743	0.413	24.763	80.060
X_3_ (Whether you have chronic diseases diagnosed by doctors)	0.598	0.458	19.940	100.000

This paper uses the weighted mean method to calculate the “health degree” of rural residents in mountainous areas of Sichuan province. The weight is the factor load of factor analysis. According to the intervals and weights of the three indicator variables, the health degree (Y_1_) obtained is calculated as shown in Equation 1.


(1) $$Y_{1}=0.472*X_{1}+0.413*X_{2}+0.458*X_{3}$$

### 2.3 The regression model

In this study, the dependent variable under scrutiny is the “health degree of rural residents,” which constitutes a subjective and objective evaluation of the health status among this population. Considering that the dependent variable and most of the independent variables in this study are continuous variables, a multiple linear regression model is employed to examine the association between the environmental literacy, control variables, and the health degree of rural residents. A multiple linear regression model is a statistical technique used to describe the relationship between two or more independent variables and a single dependent variable. It is an extension of simple linear regression, which involves only one independent variable. The equation is as follows:


(2) $$Y=\beta_{0}+\beta_{1}X_{1}+\beta_{2}X_{2}+\beta_{3}X_{3}\cdots +\beta_{j}X_{j}+\mu$$

In Equation 2, $X_{1},X_{2},\ldots ,X_{j}$ constitutes the independent variables of interest in the model, encompassing both the control variable and the environmental literacy $\beta_{0},\beta_{1},\ldots ,$ $\beta_{j}$ refers to the coefficient estimate of the influencing factor, and μ is a random error.

## 3 Result analyses

### 3.1 Descriptive statistical results

The individual and social backgrounds of rural residents encompasses nine distinct categories: gender, age, marital status, level of education, whether they are party members or village cadres, urbanization rate of the village, terrain type, and distance to the township center. Analysis of the results (Table 3) reveals that the male population constitutes the majority of rural residents, spanning from 20 to 85 years of age. A significant proportion of the populace consists of older adults individuals. Marital unions encompass 87% of the rural inhabitants, with an average educational attainment of 5.67 years, approximately corresponding to the primary school level, thus indicating a limited educational background. Membership in political parties is observed among only a small segment of rural residents, and an even smaller fraction assumes roles as village officials. In addition, concerning the control variables, 75% of the sampled rural residents reside in hilly terrain. The villages exhibit an average urbanization rate of 52.87%, denoting an overall favourable development.

**Table 3:** Descriptive statistics for scales.

		Variable	Abbreviation	Definition	Number	Mean	Standard deviation	Min	Max
Dependent variables		Health degree	HEA	Comprehensive calculation indicators	396	2.28	0.62	1.34	3.63
Independent variables	Environmental literacy	Awareness of environmental protection	AWA	1 = Strong; 0 = Weak	396	0.50	0.50	0.00	1
		Environmental protection behavior	BEH	1 = Strong; 0 = Weak	396	0.83	0.37	0.00	1
		Attitude towards environmental protection	ATT	1 = Strong; 0 = Weak	394	0.76	0.43	0.00	1
		Perception of pollution	PER	1 = Strong; 0 = Weak	392	0.25	0.44	0.00	1
	Control variables	Age	AGE	Year	396	61.54	10.82	21.00	86
		Gender	GEN	1 = Man; 2 = Woman	396	1.45	0.50	1.00	2
		Marital status	MAR	1 = Unmarried; 2 = Married	396	1.88	0.33	1.00	2
		Educational level	EDU	Year	396	5.67	3.58	0.00	15
		Party member	PAR	1 = Yes; 0 = No	396	0.15	0.35	0.00	1
		Village official	VIL	1 = Yes; 0 = No	396	0.08	0.28	0.00	1
		Terrain type	TER	1 = Mountain; 2 = Hill	396	1.75	0.43	1.00	2
		Urbanization rate	URB	Percentage	396	52.87	15.01	36.20	92.08
		Distance to the township center	DIS	km	396	4.80	3.50	0.50	14

Simultaneously, the table reveals a noteworthy pattern regarding the disposition and conduct of the residents concerning environmental protection. A majority of the residents exhibit a resolute attitude and behavior towards safeguarding the environment, while half of them display a relatively feeble awareness of environmental protection. Additionally, a considerable number of rural residents perceive a decline in pollution levels. Among the four pivotal variables under observation, the perception of pollution by rural residents emerges as the most pronounced, with a substantial portion acknowledging a decrease in pollution. On the whole, the rural residents demonstrate a certain level of environmental concern, albeit leaving room for advancement in terms of enhancing their consciousness regarding environmental protection (33).

### 3.2 The influence of rural residents’ background on the environmental literacy

To provide a comprehensive overview of the sampled rural residents and elucidate the disparities in the environmental literacy, a significant difference test was conducted on all explanatory variables. The relationship between categorical control variables and environmental literacy variables was examined using the Pearson chi-square test (χ^2^), while the association between continuous control variables and environmental variables was assessed through an independent sample *t*-test (T). The results of these statistical tests (Table 4) reveal noteworthy disparities in various aspects, including the awareness of environmental protection, among rural residents belonging to different age groups, educational backgrounds, and roles as village officials. Notably, age exhibits a significant negative correlation with awareness of environmental protection. Older rural residents encounter relatively limited avenues for accessing diverse forms of information compared to their younger counterparts, which may impede their development of a heightened consciousness towards environmental protection. Moreover, rural residents serving as village officials demonstrate significantly higher levels of awareness regarding environmental protection and perception of pollution compared to those who do not hold such positions, presumably due to the nature of their daily responsibilities. Moreover, there exists a notable positive correlation between the level of education and both awareness of environmental protection and attitude. Marital status and party membership also exert a significant positive influence on environmental behavior and perception of pollution, respectively. Unmarried rural residents display a greater inclination toward engaging in environmental practices, while farmers who hold party membership exhibit heightened concerns regarding environmental pollution, coupled with a heightened level of perception. These findings collectively underscore the noteworthy impact of rural residents’ backgrounds on the environmental literacy variables pertaining to environmental protection, a phenomenon that agrees with the theoretical framework outlined in this article.

**Table 4:** Cross-correlations of scales.

Variable	Pearson chi-square value/T value				Significance			
	AWA	ATT	PER	BEH	AWA	ATT	PER	BEH
Gender	1.4695	1.900	0.069	1.379	0.225	0.168	0.792	0.24
Age	−1.624	0.221	−0.448	1.384	0.034**	0.774	0.655	0.471
Marital status	0.2096	−0.202	−0.315	1.111	0.647	0.498	0.247	0.080**
Educational level	2.298	−0.351	0.496	−0.099	0.015**	0.058*	0.620	0.122
Health degree	1.264	2.753	0.711	−1.044	0.738	0.263	0.322	0.1995
Village official	7.438	1.427	5.823	0.954	0.006***	0.232	0.016**	0.329
Party member	1.3413	1.866	4.921	1.791	0.247	0.393	0.085**	0.408

*** means significant at the level of 1%, ** means significant at the level of 5%, * means significant at the level of 10%.

### 3.3 Impact of environmental literacy on health degree

To delve deeper into the influences exerted on the health status of rural inhabitants, an examination is conducted in this study, wherein individual background and the rural situation are introduced as control variables. Subsequently, the comprehensive effects of key observables, namely the awareness of environmental protection, attitude towards environmental protection, perception of pollution, and environmental protection behavior, on the Health degree of rural residents are explored. To accomplish this, a multiple linear regression (MLR) model is employed (Table 5).

**Table 5:** Regression results of environmental literacy’s impact on health degree.

Independent variable		Health degree						
		Model I		Model II		Model III		
		Coef. (St. Err.)	95% CI	Coef. (St. Err.)	95% CI	Coef. (St. Err.)	Standardized Coef.	95% CI
Environmental literacy	Awareness of environmental protection	0.055 (0.868)	−0.069 ~ 0.178	−0.003 (−0.049)	−0.121 ~ 0.115	0.001 (0.014)	0.001	−0.118 ~ 0.120
	Attitude towards environmental protection	0.160** (2.124)	0.012 ~ 0.307	0.161** (2.251)	0.021 ~ 0.301	0.164** (2.277)	0.112	0.023 ~ 0.304
	Perception of pollution	0.094 (1.287)	−0.049 ~ 0.237	0.091 (1.309)	−0.045 ~ 0.227	0.090 (1.297)	0.063	−0.046 ~ 0.227
	Environmental protection behavior	−0.017 (−0.206)	−0.182 ~ 0.147	−0.083 (−1.031)	−0.242 ~ 0.075	−0.080 (−0.984)	−0.048	−0.240 ~ 0.079
Control variables	Educational level			−0.008 (−0.795)	−0.028 ~ 0.012	−0.008 (−0.780)	−0.046	−0.028 ~ 0.012
	Gender			−0.131** (−1.989)	−0.260 ~ −0.002	−0.133** (−2.001)	−0.106	−0.263 ~ −0.003
	Age			−0.020*** (−6.025)	−0.027 ~ −0.014	−0.020*** (−5.879)	−0.349	−0.027 ~ −0.013
	Marital status			−0.169* (−1.839)	−0.349 ~ 0.011	−0.175* (−1.893)	−0.094	−0.357 ~ 0.006
	Village official			0.037 (0.322)	−0.190 ~ 0.264	0.023 (0.193)	0.010	−0.206 ~ 0.251
	Party member			0.315*** (3.305)	0.128 ~ 0.502	0.318*** (3.323)	0.181	0.130 ~ 0.506
	Terrain type					−0.088 (−0.903)	−0.061	−0.278 ~ 0.102
	Urbanization rate					0.003 (1.298)	0.071	−0.001 ~ 0.007
	Distance to the city center					−0.000 (−0.044)	−0.003	−0.022 ~ 0.021
	Constant	2.038*** (16.083)	1.790 ~ 2.287	3.881*** (10.209)	3.136 ~ 4.626	3.874*** (8.946)	-	3.025 ~ 4.723
Number of samples		396		396		396		
R^2^		0.022		0.141		0.148		
*F*		*F* (4,385) = 2.131, *p* = 0.076		*F* (10,379) = 6.204, *p* = 0.000		*F* (13,376) = 5.008, p = 0.000		
D-W		2.043		2.115		2.126		

**, * are significant at the levels of 1, 5%.

The regression results for the impact environmental literacy variables on the health degree of rural residents are presented in Model 1. Model 2 encompasses the regression outcomes of both the environmental literacy variables and the control variables pertaining to the characteristics of rural residents. Model 3, which combines Models 1 and 2, includes all control variables and environmental literacy variables for regression analysis. Evaluation of the pseudo R-square values reveals that Model 3 exhibits a significantly better fit than Models 1 and 2, thereby establishing it as the primary model employed for analysing the regression results in this study. Moreover, the Variance Inflation Factor (VIF) test is conducted to assess the presence of severe multicollinearity among the independent variables across different models. The VIF results demonstrate that all variables have values below 10, indicating the absence of severe multicollinearity. Furthermore, the chi-square test statistics for the models indicate that all models have successfully passed the overall significance test. This suggests that in each model, there exists a significant relationship between at least one independent variable and the dependent variable.

**Figure 3:**
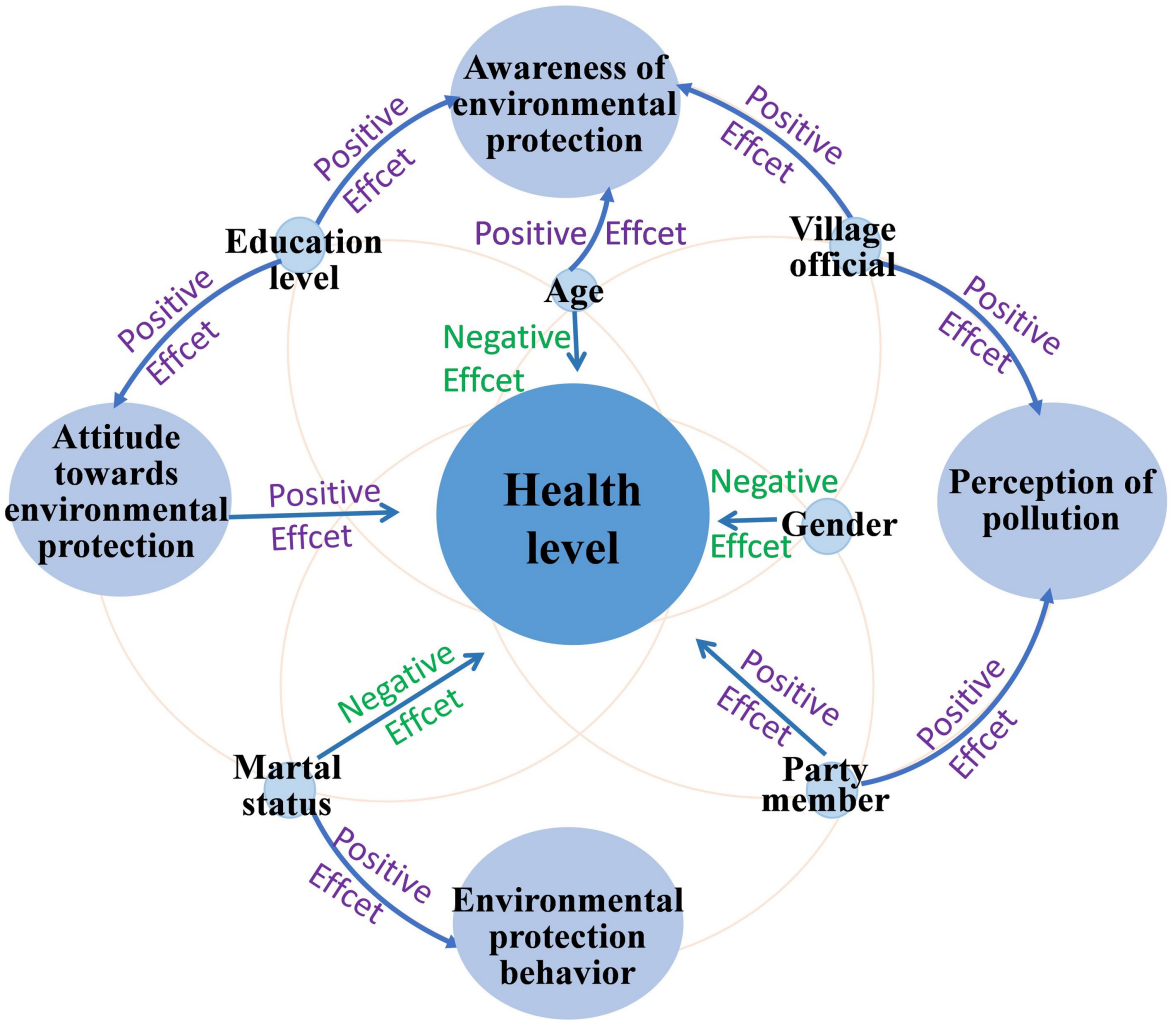
Significant influence relationship among variables.

The analysis of Model 3 regression results (Table 5) reveals a nuanced impact of the observational variables on the health degree of rural residents. And the significant influence relationship among variables is in Figure 3. A summary analysis shows that attitude towards environmental protection and being party members have a significant positive impact on the health degree. Gender, age and marital status have a significant negative impact on the health degree. However, awareness of environmental protection, perception of pollution, environmental protection behavior, educational level, assuming the role of a village official, urbanization rate, and distance to the city center do not have an impact on the health degree.

Notably, the regression coefficient value of the attitudes towards environmental protection is 0.164 (t = 2.277, *p* = 0.023 < 0.05), attitudes towards environmental protection exhibit highly significant positive impact on the health degree of rural residents. This suggests that a stronger attitudes towards environmental protection is associated with higher health degree. This implies that a one-unit increase in attitudes towards environmental protection raises the probability of rural residents opting for an average health degree by a factor of 0.164.

Consequently, rural residents who are more inclined to allocate time and financial resources towards environmental matters tend to exhibit better health conditions. Conversely, individuals who are unwilling to dedicate their time and effort to addressing environmental pollution tend to have poorer health. One possible explanation for this discovery is that rural residents with stronger environmental attitudes may be more concerned about the harmful effects of environmental pollution on their well-being, leading to higher levels of health. On the other hand, those with poorer health may exhibit lower awareness of the health consequences associated with environmental pollution. This observation aligns with existing research, which highlights that individuals dealing with health issues are often highly concerned about environmental matters (34). Similarly, awareness of environmental protection and perception of pollution also exert certain influences on the health degree of rural residents.

Among the control variables, the influence of gender, age, marital status, and being a party member on the health status of rural inhabitants is noteworthy. The impacts associated with these variables can be defined as follows: With regard to gender, male individuals residing in rural areas tend to report higher health ratings compared to those who self-assess their health as poor. Previous research findings consistently indicate that, regardless of mental or physical well-being, men generally perceive themselves as being in better health than women (35, 36). Age exhibits a significantly adverse effect on the health status of rural residents. This phenomenon can be attributed to the fact that individuals in their middle age constitute the primary workforce within their families, engaging in extensive labour and participating in numerous social activities, thereby increasing their susceptibility to injuries and illnesses. Meanwhile, the physiological functions of the older adults experience a gradual decline, accompanied by a decrease in immune response, a heightened susceptibility to functional and organic ailments, and an increased probability of accidents such as falls (37). As individuals age, their physical health progressively diminishes (38). Regarding marital status, unmarried rural inhabitants exhibit higher self-assessed health degree in comparison to those who perceive their health as poor. Moreover, assuming the role of being a party member exerts a significantly positive influence on the health status of rural residents, as they tend to report higher health ratings. In terms of the variable of education level, the data showed no significant relationship with health. The possible reason for this result is that the education level of the subjects surveyed in this paper is relatively low, which is not enough to reflect the conclusion of previous studies that the higher the education level, the more concerned about their health (39).

From Table 5, it can be seen that taking the awareness of environmental protection, attitude towards environmental protection, perception of pollution, environmental protection behavior and other control variables as independent variables, and health degree as the dependent variable for linear regression analysis, the estimated model can be:

Health Degree =3.874 + 0.001*AWA+ 0.164*ATT + 0.090*PER − 0.080*BEH − 0.008*EDU − 0.133*GEN − 0.020*AGE − 0.175*MAR + 0.023*VIL + 0.318*PAR − 0.088*LAN+ 0.003*URB − 0.000*DIS. The R-squared value of the model is 0.148, indicating that all independent and control variables can explain the 14.8% change in rural residents’ health degree.

## 4 Discussions, conclusions and implications

### 4.1 Discussions

In comparison to previous relevant studies, this study exhibits certain similarities and disparities. The selection of participants in this study predominantly focused on rural mountainous regions, and a Chinese rural resident-oriented environmental protection scale was devised. The results of this research present a more accurately depiction of the prevailing circumstances in rural mountainous areas of China.

This study highlights the positive impact of environmental protection attitudes on the health status of rural residents. Relevant studies have found that Wellbeing and quality of life are linked to a positive attitude, which makes people satisfied and healthy in their lives (40). Previous studies have shown that environmental attitude plays an important role in KAP and is the driving force of environmental behavior, but there is still a certain gap with the occurrence of behavior, which also proves the importance of environmental attitude in this result (41). At the same time, studies have proved that in the case of poor environmental awareness, there can still be a high environmental attitude, and attitude is closely related to health (42). Roy et al. (43) confirmed that the defensive attitude of the people may reduce the severity of landfill exposure, improved that defensive attitude near landfill significantly affects residents’ health status. These findings are similar to those of this study. Previous studies have often believed that psychological factors such as attitude cognition can affect health levels through behavior as a mediating variable. However, further exploration in this study found that this effect did not occur, and attitude only directly affects health levels.

In existing studies, many literatures show that attitude has a significant impact on behavior, behavior will affect health, and environment can affect residents’ health through behavioral mediators (44). However, in this paper, environmental protection attitudes directly affect health, while behaviors have no significant effect. It is speculated that this effect may be caused by the choice of behavioral variables, or it may be influenced by the complex relationship between attitude and behavior. Therefore, it is necessary to further study the relationship between attitude factors at the psychological level, behavior factors at the objective level and both subjective and objective health factors. In terms of the fundamental variables observed among rural residents, this study further reveals a robust inclination towards environmental protection, perceptiveness of pollution, and engagement in environmental behaviors. Nevertheless, the overall consciousness pertaining to environmental protection is relatively limited, which may be due to poor environmental knowledge, especially about agricultural sources of pollution (45, 46, 57). Concurrently, preceding studies have primarily emphasized the influence of factors such as age, gender, family income, educational attainment, and occupation on the awareness of environmental protection among residents (47–49). While these studies generally assert that age positively affects environmental awareness, this study also reveals a positive impact of educational level on such awareness. However, it also uncovers a negative association between age and environmental consciousness. This could potentially be attributed to the generally low educational attainment of the older adults population within the rural sample of this study, thereby resulting in an inadequate comprehension of environmental issues. Moreover, the study identifies that rural residents assuming roles as village officials exhibit a heightened awareness of environmental protection.

Previous studies on the determinants of health among rural mountain residents have predominantly focused on individual factors such as age and gender, as well as social factors such as the regional economic environment, yielding valuable insights. Qualitative studies from Western contexts have also underscored the necessary nature of environmental protection for maintaining good health (50, 51). In the findings of this study, the influence of individual factors, including gender, age, and marital status, on health degree remains notable. Some prior research suggests that a happy marriage exerts a long-lasting and stable protective effect on health. However, given that most unmarried rural residents are younger, this study reveals that they perceive their health degree as higher. The accurate mechanism by which marital status operates necessitates further research. Moreover, the roles of party members also emerge as significant in this study. Notably, this article categorizes the sampled rural areas residing in mountainous regions based on terrain, uncovering substantial disparities in the health degree of residents between mountainous and hilly villages. The terrain factor warrants further exploration in future research.

### 4.2 Conclusions and policy recommendations

This study employs field research data to scrutinize the influence of geographical conditions prevalent in mountainous rural regions, the degree of environmental literacy exhibited by rural inhabitants, and various other factors on the overall health status. A comprehensive scale is devised to assess rural residents’ environment literacy, which includes cognizance of environmental preservation, their attitudes towards it, corresponding behaviors, and perception of pollution. Additionally, aKAP model, encompassing both environmental literacy and control variables, is constructed to elucidate the relationship between rural resident health and the aforementioned variables. Comparative to extant research, this study accentuates the potential repercussions stemming from environmental literacy and other environmental factors on the health status of rural residents, leading to the formulation of the following conclusions:

1. Environmental literacy of rural residents is significantly influenced by demographic factors. Age shows a negative correlation with environmental protection awareness, while education level and village official status are positively associated. Education is also linked to more positive environmental attitudes, and marital status influences environmental behaviors, with unmarried individuals being more proactive. Party membership correlates with greater concern for pollution.
2. The impact of environment literacy on rural residents’ health level is complex. Positive attitudes towards environmental protection are significantly linked to better health status, and increased awareness and pollution perception contribute to improved health.
3. Among control variables, age, marital status, gender, and party membership significantly affect health. These findings inform more scientific and effective approaches to improving rural health.

This study presents a novel vantage point regarding the investigation of environmental literacy and the state of health. Moreover, it evaluates, to a certain extent, the efficacy of policies associated with ecological civilization. This indirect inquiry sheds light on the local residents’ endorsement of environmental policies and support for related actions, while simultaneously providing a direct glimpse into the environmental consciousness, attitudes, behaviors, and perceptions of pollution held by the aforementioned rural dwellers. Drawing upon the empirical findings, the following recommendations are proffered: It is necessary for relevant governmental bodies and organizations to fortify environmental conservation education and technical training initiatives targeted at rural regions. Additionally, incentivizing eco-friendly behaviors and fostering the enhancement of educational attainment among residents of the mountainous rural areas should be prioritized. The media and propaganda departments should actively engage in diverse environmental conservation training programs and dissemination of information, emphasizing the criticality of cultivating environmental literacy for the betterment of the rural ecosystem and individual well-being. Within this context, rural party members and officials ought to assume a pioneering and leading role to elevate rural residents’ cognizance of environmental protection and encourage a heightened focus on personal health status.

## 5 Limitations

This study is not without certain limitations that warrant acknowledgement and further exploration. Firstly, it is necessary to recognize that the research was conducted exclusively within a representative mountainous region of Sichuan. Although data acquisition was carried out via random sampling to mitigate sampling bias, there may still exist isolated cases that deviate from the characteristics elucidated in this paper. Secondly, this paper also identifies avenues for potential advancement in future studies. At the same time, due to the reality that the existing rural residents in mountainous areas inevitably have a large proportion of older adults people and low education level, the different results of the research conclusions are also more targeted. For instance, the selection of indicators for control variables remains incomplete, and the inclusion of factors related to natural disasters could enhance the comprehensiveness of the assessment system. In addition, the analysis of the causal mechanisms between control variables and rural environmental results can be fortified. Thirdly, it is essential to recognize that the health status of rural inhabitants is a dynamically developing indicator. In future studies, there is potential for tracking data over subsequent years to facilitate a more comprehensive analysis of the long-term dynamics and impact mechanisms, thereby yielding more valuable insights for enhancing the health status and environmental consciousness of rural inhabitants. Moreover, given the perennial concern surrounding health-related matters, this paper substantiates a certain correlation between attitudes towards environmental protection, environmental preservation behaviors, and health degree. Consequently, further investigations should seek to address pertinent questions such as the effective methods to enhance environmental preservation behaviors and health degree among rural residents in mountainous regions, as well as the development of robust evaluation frameworks for assessing their health degree.

## Data Availability

The raw data supporting the conclusions of this article will be made available by the authors, without undue reservation.
